# Construction and Validation of an Anticipatory Thinking Assessment

**DOI:** 10.3389/fpsyg.2019.02749

**Published:** 2019-12-11

**Authors:** Michael Geden, Andy Smith, James Campbell, Randall Spain, Adam Amos-Binks, Bradford Mott, Jing Feng, James Lester

**Affiliations:** ^1^Center for Educational Informatics, North Carolina State University, Raleigh, NC, United States; ^2^Laboratory for Analytic Sciences, North Carolina State University, Raleigh, NC, United States; ^3^Applied Research Associates, Raleigh, NC, United States; ^4^Department of Psychology, College of Humanities and Social Sciences, North Carolina State University, Raleigh, NC, United States

**Keywords:** anticipatory thinking, prospective cognition, divergent thinking, assessment development, validation

## Abstract

Anticipatory thinking is a critical cognitive skill for successfully navigating complex, ambiguous systems in which individuals must analyze system states, anticipate outcomes, and forecast future events. For example, in military planning, intelligence analysis, business, medicine, and social services, individuals must use information to identify warnings, anticipate a spectrum of possible outcomes, and forecast likely futures in order to avoid tactical and strategic surprise. Existing methods for examining anticipatory thinking skill have relied upon task-specific behavioral measures or are resource-intensive, both of which are challenging to scale. Given the increasing importance of anticipatory thinking in many domains, developing a generic assessment of this skill and identifying the underlying cognitive mechanisms supporting it are paramount. The work reported here focuses on the development and validation of the anticipatory thinking assessment (ANTA) for measuring the divergent generative process of anticipatory thinking. Two-hundred and ten participants completed the ANTA, which required them to anticipate possible risks, opportunities, trends, or other uncertainties associated with a focal topic. Responses to the anticipatory thinking and divergent thinking tasks were rated by trained raters on a five-point scale according to the uniqueness, specificity, and remoteness of responses. Results supported the ANTA’s construct validity, convergent validity, and discriminant validity. We also explored the relationship between the ANTA scores and certain psychological traits and cognitive measures (need for cognition, need for closure, and mindfulness). Our findings suggest that the ANTA is a psychometrically valid instrument that may help researchers investigate anticipatory thinking in new contexts.

## Introduction

Anticipating emergent threats, future events, and consequences of events requires individuals to proactively consider and make sense of many dynamic components of complex systems and situations. This cognitive process, anticipatory thinking, can be conceptualized as the deliberate exploration and analysis of relevant alternative system states. Anticipatory thinking is related to but distinct from prediction (Klein et al., 2007) and relies on many connected cognitive components including attention, memory, executive function, situation awareness, and domain expertise (Koziol et al., 2012; Mullally and Maguire, 2014). While there is no consensus on a formal definition of anticipatory thinking, leaders in business, military, and the intelligence community recognize the importance of anticipatory thinking and its value in solving complex problems, strategic foresight, and strategic planning (Flournoy and Brimley, 2006; Hines and Bishop, 2006; Fuerth, 2009; Anderson, 2011). In these critical task domains, anticipatory thinking enables analysts and decision makers to imagine a range of possible futures and identify indicators that could lead to these future states. In these domain-specific tasks, anticipatory thinking is generally purposive and often constrained by the real-world context. Both deliberate and spontaneous thought processes may be involved in anticipatory thinking.

**FIGURE 1:**
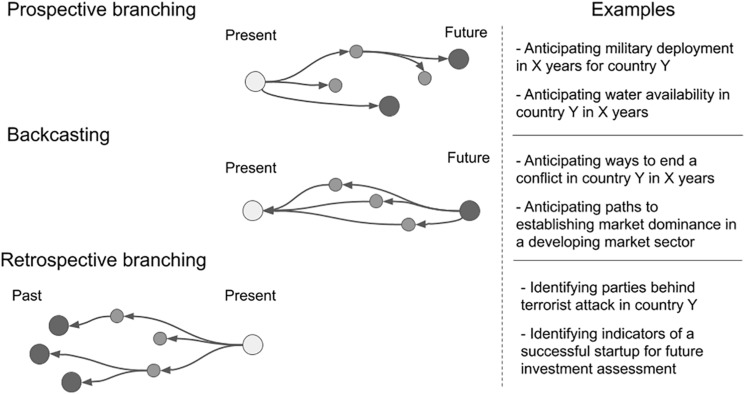
Types of anticipatory thinking. Light gray circles represent the present, medium gray represent intermediate states, and dark gray represent states of interest. Arrows depict the direction in which the analyst is anticipating.

Anticipatory thinking can take three distinct forms; prospective branching, backcasting, and retrospective branching (Figure 1). *Prospective branching* involves anticipating future system states and identifying indicators that may lead to these system states. *Backcasting* involves examining a particular future system state and thinking back in time to identify warnings and indicators that lead to its occurrence. *Retrospective branching* is the identification of possible unknown past system states and their paths toward the present one. Examples of each of these forms of anticipatory thinking are presented in Figure 1. All forms of anticipatory thinking focus on the mapping of alternative system states and paths toward them through uncertain conditions, and the goals of the analyst influences where the uncertainty is being mapped out.

Each form of anticipatory thinking involves three distinct processes; *recognition* of a situation based on current cues derived from previous experience, *extrapolation* of a system state to a different state, and *construction* of a mental model of the system based on variable evidence (Klein et al., 2007; McLennan et al., 2009). Extrapolation is particularly important when navigating the large set of state spaces present in ambiguous and complex systems as it generates a corpus of system states that can then be used for recognition and construction. According to Mumford et al.’s (1991) creative process model, the extrapolation phase of anticipatory thinking is likely to include a convergent thinking component in which potential future states approach an ideal solution path and a divergent phase in which many possible futures are generated in conjunction to produce creative ideas (Cropley, 2006). There has been some work explicitly supporting the link between the extrapolation component of anticipatory thinking to creativity. Osburn and Mumford (2006) found that forecasting led to “higher quality and more original” problem solving. Additional evidence was provided by Byrne et al. (2010), who found that anticipating the implication of advertising ideas and plans led to more creative problem solving, and that simulation extensiveness had a strong predictive effect on idea quality.

Measuring anticipatory thinking is particularly challenging because the process does not directly result in the creation of measurable artifacts. The few existing methods for examining anticipatory thinking have relied upon task-specific behavioral measures (Doane et al., 2004; Rosalie and Müller, 2013) or resource-intensive interviews (McLennan et al., 2009), both of which are difficult to scale. A generic assessment would make measurement of, and research on, anticipatory thinking easier and provide potential utility in the training, selection, and hiring of analysts and other individuals who engage in risk assessment and forecasting activities. The primary goal of this paper was to construct a generalizable, less resource intensive instrument for measuring the skill of anticipatory thinking, the anticipatory thinking assessment (ANTA), and to evaluate the validity of the new instrument.

A secondary goal of the work described in this article was to use the ANTA to explore the relationship of anticipatory thinking with several dispositional factors. Mellers et al. (2015) provide an account for how individual characteristics and dispositional factors could influence anticipatory thinking in intelligence analysis, a field that heavily employs anticipatory thinking. The current study explores three potentially relevant dispositional factors; need for closure, need for cognition, and mindfulness. *Need for closure* is the discomfort that an individual experiences with ambiguous situations (Webster and Kruglanski, 1994), which would be expected to have a negative relationship with anticipatory thinking due to its fundamentally ambiguous nature. *Need for cognition* is the motivation that an individual has for mentally stimulating activities (Cacioppo and Petty, 1982). It has a positive relationship with more extensive information search behaviors (Verplanken et al., 1992) and should be positively correlated with anticipatory thinking since it is a mentally demanding process involving extensive information gathering to develop an accurate understanding of the current and plausible alternative system states. Both need for cognition and need for closure are also related to openness to experience (Sadowski and Cogburn, 1997; Onraet et al., 2011), which has been found to correlate with creativity and divergent thinking (McCrae, 1987). *Mindfulness* is the tendency to be attentive and open-minded in the present (Bishop et al., 2004) and is associated with increased creativity through improved perspective switching and creative elaboration (Lebuda et al., 2016). Anticipatory thinking requires the creative generation of alternative system states and would be expected to be positively correlated to mindfulness.

## Theoretical Background

### Imagining Alternative Realities

Anticipatory thinking requires imagining beyond the present system state to alternative system states at a different time point. This imagination can be considered as a form of mental time travel (Tulving, 2002), wherein individuals mentally simulate novel events through either the replay of existing semantic (Irish et al., 2012) and episodic memories (Addis et al., 2007) or the unique recombination of them to imagine alternative past or future events (Suddendorf and Busby, 2005). According to the theoretical account of mental time travel, imagining past and unrealized events share an underlying mechanism, a claim which has received support from numerous neurological studies (Okuda et al., 2003; Schacter et al., 2007, 2008; Botzung et al., 2008).

More generally, future-oriented mental time travel is one topic belonging to the broader field of prospection, which investigates how people look into the future. Szpunar et al. (2014) proposed a taxonomy for the diverse field of prospective cognition using either semantic or episodic memories across four separate modes; simulation of events through recombination of memories (e.g., imagine events that could happen at a future meeting), prediction of future events (e.g., determine whether the meeting will go well), identification of the intentions of involved agents (e.g., considering the goal of the meeting team), and planning by utilizing this information (e.g., organizing steps to prepare for the future meeting). Within the context of anticipatory thinking, we are primarily interested in simulation of events and identification of intentions, as anticipatory thinking does not include planning and it extends beyond predicting the most likely state. Prospective branching and backtracking are cyclical processes of simulating a suite of alternative system states, interpreting the intentions of relevant parties in those states, and inferring subsequent plausible branching states. The traversal of one alternative system state to another likely employs the use of conditional reasoning, a form of logical reasoning using if-then statements (e.g., if the ice caps melt, then water levels will rise), which is thought to be a required component for engaging in prospection (Seligman et al., 2013).

The goal of anticipatory thinking is to generate a range of possible future or past system states that can be interpretable and useful. The complexity of real-world problems prevents the analyst from simply decomposing every state into facets and exploring every combination, as this would result in an excessively large space of system states, many of which would not be of interest or sensible. It would also induce a tremendous amount of cognitive load that is beyond an analyst’s limited working memory capacity. Instead, the analyst engages in a creative process only considering useful and plausible states. Creativity within the real-world problem space requires a certain degree of domain-specific expertise (Baer, 2015), as expertise is essential in recognizing the value of an idea and in providing a body of knowledge that can be recombined in novel ways.

### Anticipatory Thinking in the Field

Analysts employ a number of structured analytical methods to support anticipatory thinking, often as part of a comprehensive program to provide warnings or indicators of critical change. For example, two common methods are scenario analysis and futures wheel. *Scenario analysis*, or *alternative futures analysis*, is a group-based method involving the selection of an outcome or goal, indicators of that goal, identification of predictable and unpredictable forces of change, and the discrete mapping of the uncertainties onto a small set of detailed scenarios (Schwartz, 1991). The identification of indicators and uncertainties provides a flexible base for anticipatory thinking, and there has been some research tying it to prospective thinking (McKiernan, 2017). Scenario planning works well for ill-defined problems due to its flexibility, but is time-consuming to conduct and difficult to assess.

*Futures wheel* is a graphical team-based structured analytic method involving mapping out consequences to a particular event (Glenn, 1972). After generating the first-order consequences, the process can be repeated by treating each first-order consequence as the event and mapping out additional second-order events. This process can be repeated any number of times, but is generally stopped at third-order consequences to keep the futures wheel at a manageable size. The wheels can be analyzed by having the team rate responses in terms of their desirability and likelihood (Schreier, 2014) or by inductive thematic analysis (Benckendorff, 2008). Futures wheel is a convenient method due to the speed of collection and is commonly used in educational settings (BouJaoude, 2000), but it becomes unmanageably large when the primary event is ill-defined as it is in many real world situations. An example report utilizing futures wheel can be found in Heinonen et al. (2017).

Other related methods, such as outside-in thinking, structured brainstorming, and backcasting can be used in combination with scenario, alternative futures, and future wheels analysis as part of long-term analytic efforts (United States Government [USG], 2009). As an example of the result of a comprehensive anticipatory thinking analysis, the World Economic Forum (2017) provided a detailed case study applying anticipatory thinking to the question of “how the food system will nutritiously and sustainably feed 8.5 billion people in 2030.” A panel of experts used scenario analysis to identify key indicators, uncertainties, and trends which resulted in the construction of four relevant scenarios. Similarly, scenario analysis was also used to investigate the future of water in Egypt for 2025 (Shakweer and Youssef, 2007).

### Development of Anticipatory Thinking Assessment

A consistent theme that emerges from the cognitive science literature is the vital role of the generative process in anticipatory thinking, a component of anticipatory thinking that enables successful generation of a space of alternative system states. Assessing this process, however, can be challenging. Existing approaches rely on resource-intensive interviews about previous experiences, which are primarily informative for the recognition and construction components of anticipatory thinking, but provide limited insight on the extrapolation component. These interviews require domain expertise to evaluate and do not readily extend to other contexts. To address these challenges, we developed a novel ANTA technique that aimed to engage individuals in the extrapolation and construction components of anticipatory thinking. A central goal in creating the ANTA was to ensure that the assessment method was easy to modify for different domains, easy to administer, and that the outcomes could be quantitatively examined. We used scenario analysis as the basis of the ANTA due to its flexibility and widespread use; however, we made several changes to the methodology to meet the goals outlined above.

The ANTA requires individuals to read a target statement about a future event (e.g., “how the food system will nutritiously and sustainably feed 8.5 billion people in 2030”) and then produce as many pairs of potential future events (i.e., uncertainties) and their subsequent impact as possible within a predetermined time frame. Uncertainty–impact pairs are elicited in lieu of simply stating the alternative state (impact) as it closely mirrors the natural conditional logic used in prospection (if A then B) and it discourages responses that cannot be connected to previous states through some predicate. This simple methodology allows a great deal of flexibility. First, the pairs can be stacked to create *n*th order consequences as seen in futures wheel through mapping the impact of a previous pair as the uncertainty of a subsequent pair (for a description of futures wheel, see the section “Anticipatory Thinking in the Field.”). As an example, for the target statement feeding the world’s population in the future (Table 1), one uncertainty could be a potential rapid adoption of new food technology and its impact could be the population’s increased preference for vegetarianism (World Economic Forum, 2017). This impact in Pair 1 becomes the uncertainty in Pair 2, with the impact being lower resource requirements for production. Second, the same uncertainty can result in multiple impacts across different pairs. Again, using the case of feeding the world’s population as an example (Table 1), large-scale warfare serves as the uncertainty in both Pair 3 and Pair 4, with a different impact listed in each pair. Despite the flexibility and simplicity of the ANTA, the modifications from scenario analysis came with a cost; the ANTA loses much of the fine-grained detail of scenario analysis, such as being unable to effectively handle interactions of uncertainties (e.g., a joint uncertainty of market connectivity and resource consumption; World Economic Forum, 2017). Other important limitations of the ANTA include its severe time limit, in contrast to much anticipatory thinking in support of strategic analysis, and individual work. We considered these limitations to be acceptable and necessary for an efficient assessment, because the goal of the ANTA was not to replace the currently existing methodology but to provide a new method that can be used for assessing an analyst’s skill in anticipatory thinking.

**TABLE 1:** Example ANTA responses (four uncertainty–impact pairs) for feeding 8.5 billion people in 2030 (World Economic Forum, 2017).

**Pair #**	**Uncertainty**	**Impact**
1	Rapid adoption of new food tech	Increased preference for vegetarianism
2	Increased preference for vegetarianism	Lower resource requirements for production
3	Large-scale warfare	Wartime costs raise production costs decreasing food availability
4	Large-scale warfare	Use of modern weapons destroys arable land, reducing food production

*Each row represents a single uncertainty and impact pair that a participant could provide.*

Measuring anticipatory thinking from the ANTA requires identifying characteristics of good anticipatory thinking. From reviewing two detailed case studies (Shakweer and Youssef, 2007; World Economic Forum, 2017) as well as analytical guidance for anticipatory thinking (Grabo, 2002; United States Government [USG], 2009), we identified four qualities of anticipatory thinking; the detection of low-probability/high-impact events, unique implications of uncertainties, detailed elaboration, and the diverse coverage of alternative states. These qualities were, respectively, mapped onto four ratings with the ANTA; *uniqueness*, *remoteness*, *specificity*, and *diversity* (Table 2).

**TABLE 2:** ANTA ratings.

**Rating**	**Description**
Uniqueness	The level of uniqueness of the response
Remoteness	The creativity of the uncertainty and impact pair
Specificity	The level of elaboration of the response
Diversity	The breadth of ideas spanned across all of a participant’s responses

The first two components (*uniqueness* and *remoteness*) were created from a modification of the measures used for divergent thinking from Silvia et al. (2008), which were adapted from Wilson et al. (1953). We developed two prompts for the ANTA: (1) *The impact of smart home technologies on older adults in 10 years* and (2) *The availability and use of leisure time in 10 years*. The content validity of the ANTA was informally assessed by presenting it to several subject matter experts (SMEs) in the intelligence community. They confirmed that, while the above-mentioned limitations to assessment make it partially removed from practice, the assessment reflects critical aspects of successful anticipatory process and output.

## The Present Study

In the following section, we report the results of a study evaluating the construct, convergent, and discriminant validity of the proposed ANTA. The study involved administering the ANTA, several divergent and convergent thinking tasks, and a survey of other measures that were hypothesized to be related to performance on the ANTA to a large sample of participants. Responses to the ANTA were reviewed by trained raters and assessed according to the four ratings described above. These outcomes were then examined against performance on the divergent and convergent thinking tasks to evaluate the ANTA’s validity.

## Materials and Methods

### Participants

All participants (*n* = 210; 70 men, 140 women) were recruited from Amazon’s Mechanical Turk with a compensation of $2.50 for completing the study. There was a wide distribution of ages across participants (*M* = 37.88 years, *SD* = 12.9, minimum = 18, maximum = 85). Participants were required to have a 95% Human Intelligence Task (HIT) approval rate, be l8 years of age or older, reside in the United States, and to indicate English as their primary language in order to be eligible for the study.

### Instruments

#### Anticipatory Thinking Assessment

The ANTA utilized two different prompts. The first asked respondents to anticipate the impact of smart home technologies on older adults in 10 years; the second prompted participants to think about the availability and use of leisure time in 10 years. The prompts were selected to address general topics and posed with a long-enough timeframe that technical or domain expertise would not dominate responses. Participants were given 10 min to generate as many pairs of uncertainties and impacts as they were able to for each prompt. Participants were told that the goal was to “provide creative and unique responses that describe as many possible futures as you can.” This instruction emphasizing the creativity and uniqueness of responses directly corresponds to the four ratings. Similar instruction has been recommended by prior literature on divergent thinking (Harrington, 1975; Silvia et al., 2008). Examples of strong and weak responses were given during the instruction phase. The time frame specified in each prompt (i.e., 10 years) was selected after piloting the tasks with the research team and SMEs. The instruction for participants to complete the ANTA is provided in Supplementary Appendix A.

Responses were assessed by three trained raters using three qualitatively coded ratings; uniqueness, specificity, and remoteness. *Uniqueness* is the originality of the response, *specificity* is the amount of elaboration for the response, and *remoteness* is the creative linking in the pairing between the uncertainty and impact (the scoring of *diversity* is discussed below). Each rating was on a scale of 1–5, with 1 indicating an answer that is unoriginal/common/unspecific and 5 being original/unique/elaborated. Uniqueness and specificity were rated for each individual uncertainty and impact response, generating two ratings for each uncertainty–impact pair (one for uncertainty, one for impact). Remoteness was rated for each pair as a whole, and only produced one response for each uncertainty–impact pair. Each participant’s score was calculated as the average of their top two responses so as to prevent people from being punished for exploring many of the non-unique possibilities.

The raters also assigned each uncertainty or impact response to a specific category. Categories were formed separately for each of the prompts through a grounded theory approach (Strauss and Corbin, 1994) based around PESTLE (political, economic, socio-cultural, technological, legal, and environment). The research team first reviewed PESTLE as a springboard and then labeled each uncertainty or impact response with a label that they thought was appropriate. All of the labels were then combined and clustered into a smaller set of categories. These categories were tested on the pilot data, re-evaluated, and finalized (see Table 3).

**TABLE 3:** Categories of responses for the ANTA prompts.

**Prompt**	**Categories**
Smart home	Demographics/individual characteristics, economics (macro), emotional health, environmental, health—physical/other, mobility, political/legal, security/privacy/malicious use, social norms/way of life, technology—adoption/trust, technology—cost, technology—quality/capability, usability
Leisure time	Economics (macro), entertainment/leisure time, food, global health (environmental), law/crime, mobility, physical health, political, social, technology, work

Finally, respondents were assessed based on the total diversity of responses. Diversity was calculated separately for uncertainties and impacts based on the total number of categories for which the respondent provided at least one response. The diversity metric shared some similarity to a measure commonly used in divergent thinking literature called fluency, which is the number of responses that a participant generates. There have been well-documented concerns raised about the use of additive creativity scores and fluency due to common method bias; however, we take an approach described in Reiter-Palmon et al. (2019) to circumvent this issue by taking the average of the top two scores for our subjective rating metrics (i.e., specificity, novelty, remoteness). The instruction for raters to rate ANTA responses is provided in Supplementary Appendix B.

#### Convergent Validity

Based on a literature review of psychological constructs relevant to anticipatory thinking, two constructs were selected for establishing convergent validity: divergent thinking and convergent reasoning. *Divergent thinking* is expected to be a necessary component of anticipatory thinking in the creation of alternative system states. Divergent thinking was measured using the methods outlined in Silvia et al. (2008) through combining results from an unusual uses task, an instances task, and a consequences task. *Convergent reasoning* allows for the interpolation between alternative system states and was measured using the 3-min verbal reasoning assessment developed by Baddeley (1968). Unfortunately, due to a systematic error in the experiment software configuration, the 3-minute timer was not enforced.

#### Discriminant Validity

The two instruments selected for establishing discriminate validity for the ANTA measured loneliness (Cronbach’s α = 0.87) and well-being (α = 0.91). The loneliness scales (Hughes et al., 2004) included three items. The well-being scale (Diener et al., 2009) had eight items. These two instruments were selected based on the assumption that an individual’s general loneliness and well-being should not relate to anticipatory thinking skills.

#### Dispositional Factors

Need for cognition (α = 0.94) was measured using the 18-item scale developed by Cacioppo et al. (1984). Need for closure was measured using the 15-item measure developed by Roets and Van Hiel (2011). The scale has five subscales; order (α = 0.90), predictability (α = 0.83), decisiveness (α = 0.70), ambiguity (α = 0.77), and closed-minded (α = 0.61). Mindfulness was assessed using the 39-item measurement developed by Baer et al. (2006) the measures the general tendency to be attentive to and aware of present-moment experience in daily life. The mindfulness instrument has five subscales; observe (α = 0.85), describe (α = 0.88), awareness (α = 0.91), non-judge (α = 0.91), and non-react (α = 0.82).

### Qualitative Coding

Responses to the anticipatory thinking and divergent thinking tasks were evaluated by three raters per task in a saturated fully crossed design. Raters were trained for each task by practicing on pilot data generated from the research team. The first step in both tasks was to read all of the responses before doing any ratings in order to get a sense of how common certain ideas were. The divergent thinking task data were presented to raters in a random order wherein they rated the uncommonness, cleverness, and remoteness of each response according to a protocol adapted from Silvia et al. (2008). For the ANTA, coders first categorized each individual impact and uncertainty as a group with the data in a randomized order. Responses that were uninterpretable were coded as an N/A and were not counted in the calculation of the diversity score. After categorization, each coder individually went through one category at a time reading all of the responses and then rating each response on its uniqueness, specificity, and remoteness. Each task had the same set of raters throughout all the participants except for the ANTA leisure time prompt, which required two sets of three raters as the first set were unable to complete rating all responses. Interrater reliability for the anticipatory thinking and divergent thinking tasks for each rating was calculated using kendall’s tau (see Table 4).

**TABLE 4:** Interrater reliability table (Kendall’s tau).

**ANTA**			**Divergent thinking tasks**			
**Rating**	**Smart home**	**Leisure time**	**Rating**	**Unusual uses**	**Instances**	**Consequences**
*N*	5942	8030	*N*	2019	1481	1624
Remoteness	0.92	0.93, 0.79	Remoteness	0.57	0.63	0.44
Uniqueness	0.73	0.83, 0.79	Uncommonness	0.56	0.76	0.48
Specificity	0.82	0.89, 0.86	Cleverness	0.33	0.72	0.39

*Leisure time has two ratings because two different sets of raters were used. *N* represents the number of responses generated. Uniqueness and specificity for the ANTA were conducted for each impact/uncertainty giving them a sample size of 2*N*.*

### Procedure

After agreeing to enroll in the study, participants were directed to an informed consent survey hosted on Qualtrics and required to accept in order to continue with the study. All activities were administered electronically using Qualtrics. The first activity that participants completed was a practice abbreviated version of the ANTA on the World Economic Forum (2017) topic in which they were provided with some examples and given three minutes to complete the task. After the training, participants were given the two full ANTA tasks in a randomized order. Following the ANTA participants completed the other instruments in a randomized order, with all demographic questions at the end of the study. The experiment in total took approximately 45 min to complete.

## Results

Data were cleaned using R (R Core Team, 2018) and python. All analyses were conducted in R. The confirmatory factor analysis (CFA) was run using the lavaan package (Rosseel, 2012). Similar numbers of response pairs were generated for both the leisure time task (*M* = 24.31, *SD* = 9.62) and the smart home task (*M* = 23.83, *SD* = 10.14).

### ANTA Factor Structure and Internal Consistency

An individual’s scores on the ANTA metrics (diversity, specificity, novelty, remoteness) were constructed through averaging across the top two responses along all three raters for both the leisure time and smart home tasks. This method was adapted from Silvia et al. (2008) in order to prevent punishing individuals who generated a large number of low-creativity responses. Metric invariance was assessed in order to evaluate the appropriateness of combining the results of both tasks into a single set of scores for the CFA and multiple regression. There were mixed results in the difference of goodness-of-fit between the unconstrained and constrained models [Δ^2^(3) = 13.67, *p* = 0.003, ΔSRMR = 0.035, ΔRMSEA = 0.105, ΔCFI = −0.008, ΔAIC = 7.676, ΔBIC = −4.445]. The RMSEA is included but should be interpreted with caution due to its inflation with low degrees of freedom models (Kenny et al., 2015). The standardized factor loadings for the unconstrained model were similar for uniqueness, specificity, remoteness, and diversity across the smart home (0.88, 0.95, 0.92, 0.54) and leisure time tasks (0.94, 0.92, 0.89, 0.57). Correlations across both tasks for each of the metrics were moderate to high as well (*r*_remoteness_ = 0.83, *r*_uniqueness_ = 0.97, *r*_specificty_ = 0.83, *r*_diversity_ = 0.66). Diversity exhibited the largest difference due to the different total number of categories across the two tasks; however, considering the similarity of the standardized factor loadings, these differences were considered minor enough for aggregation across tasks to be appropriate.

The four-item structure for the ANTA (uniqueness, specificity, remoteness, and diversity) was tested through CFA using maximum likelihood estimation. The goodness-of-fit metrics suggested a strong model fit [Δ^2^(2) = 7.54, *p* = 0.023, SRMR = 0.017, RMSEA = 0.115, CFI = 0.993]. Diversity (standardized 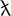 0.59, *p* < 0.001), specificity (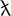 0.95, *p* < 0.001), novelty (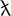 0.95, *p* < 0.001), and remoteness (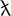 0.93, *p* < 0.001) all had significant factor loadings on the anticipatory thinking construct. All four items of the ANTA displayed an acceptable internal consistency with a Cronbach’s alpha of 0.75. There were high correlations between the three creativity scales (Specificity, Uniqueness, and Remoteness) and a moderate correlation with diversity (see Table 5).

**TABLE 5:** ANTA descriptive statistics.

**Facet**	***M***	***SD***	**1**	**2**	**3**	**4**
Remoteness	3.24	0.85	1			
Uniqueness	3.06	0.79	0.88	1		
Specificity	3.09	0.77	0.88	0.91	1	
Diversity	6.87	2.43	0.59	0.56	0.88	1

A single ANTA score is then calculated through averaging an individual’s scores across the four different ANTA metrics. Table 6 shows the correlations across the ANTA score and the convergent validity, discriminant validity, and dispositional variables.

**TABLE 6:** Correlation matrix.

**Variable**	**1**	**2**	**3**	**4**	**5**	**6**	**7**	**8**
1. ANTA	1.00							
2. Convergent thinking	0.39^∗∗∗^	1.00						
3. Divergent thinking	0.53^∗∗∗^	0.22^∗∗^	1.00					
4. Need for cognition	0.12	0.18^∗^	0.18^∗^	1.00				
5. Need for closure	–0.03	–0.01	–0.07	–0.38^∗∗∗^	1.00			
6. Mindfulness	0.05	0.16^∗^	0.13	0.48^∗∗∗^	–0.18	1.00		
7. Flourishing	0.01	–0.05	0.06	0.34^∗∗∗^	–0.04	0.57^∗∗∗^	1.00	
8. Loneliness	0.11	0.04	0.07	−0.12^∗^	0.13^∗^	–0.54^∗∗∗^	–0.55^∗∗∗^	1.00

**^∗^p* < 0.05, ^∗∗^*p* < 0.01, ^∗∗∗^*p* < 0.001.*

### Convergent Validity, Discriminant Validity, and Relation to Other Dispositional Factors

A single multiple linear regression was run with all personality measures, convergent constructs, and divergent constructs. Scales were constructed as the mean of all their corresponding items. None of the personality measures had a significant relationship with the ANTA (see Table 7). Both convergent validity constructs, convergent reasoning and divergent thinking, were significantly positively related with the ANTA score. Neither of the discriminant validity constructs, loneliness and well-being, had a significant relationship with the ANTA.

**TABLE 7:** ANTA linear regression results.

**Construct**	***b***	**SE**	***p***
**Convergent validity**			
Convergent reasoning	1.59	0.31	<0.001
Divergent thinking	0.85	0.11	<0.001
**Discriminant validity**			
Loneliness	0.13	0.12	0.293
Well-being	0.08	0.08	0.290
**Psychological traits**			
Need for closure	–0.01	0.15	0.844
Need for cognition	–0.02	0.15	0.906
Mindfulness	–0.12	0.15	0.423

*All results are from the same model. Unstandardized weights are used due to non-normality in some of the covariates which can produce misleading interpretation when comparing variables.*

## Discussion

Anticipatory thinking allows individuals to effectively navigate through an uncertain world and is a critical component in a variety of fields such as risk assessment, business management, and intelligent analysis. Research on anticipatory thinking has been limited due to the lack of a flexible and easy-to-deploy instrument. The primary goal of the present study was to develop and validate a domain-adaptable instrument for measuring the divergent component of anticipatory thinking, with particular focus on the extrapolation and construction facets. The four components of the proposed ANTA were the *diversity* of the explored space, the *specificity* of the idea, the *novelty* of the idea, and the *remoteness* of the uncertainty–impact pair. All components significantly loaded on the latent factor with moderate fit on the CFA. The ANTA displayed good convergent validity, discriminant validity, and internal consistency. The three ANTA creativity scores based on Silvia et al. (2008)’s method were highly correlated, suggesting that creative anticipatory thinking ideas typically entail all three characteristics.

The secondary goal of the study was to apply the ANTA in order to explore its relationship with three dispositional factors; need for cognition, need for closure, and mindfulness. Need for cognition and need for closure were thought to provide internal motivation (or depletion) for sustained engagement in the challenging process of anticipatory thinking, while mindfulness was hypothesized to be related to the deliberate process of anticipatory generation. Prior research showed a positive connection between need for cognition and curiosity, engagement in intellectual activities, as well as openness to ideas (e.g., Berzonsky and Sullivan, 1992; Mussel, 2010), all of which are highly relevant to openness of experience, the personality trait among the Big Five that is most predictive of creativity (Oleynick et al., 2017). More direct evidence has been obtained that need for cognition is positively related to creative problem solving as measured by strategic planning to achieve organizational goals (Watts et al., 2017). In contrast, need for closure has been typically found to limit creative performance and generation of alternative solutions (e.g., Chirumbolo et al., 2004). Practice of mindfulness and meditation was found to enhance divergent thinking (Colzato et al., 2012; Ostafin and Kassman, 2012). However, unlike what we expected, none of the dispositional factors had a statistically significant relationship with the ANTA. One potential explanation could be that the motivational differences seen in need for cognition and need for closure may be obfuscated by the external motivation of the monetary reward within the MTurk sample. A second explanation could be that these psychological traits influence the anticipatory thinking process only after initial ideation saturation, which is not being reached in the abbreviated form of the ANTA. A task such as extensive scenario planning that requires more elaborated anticipatory responses may show performance that is more strongly connected with these traits. The third explanation could be that none of these traits as captured by the current measures relate to anticipatory thinking. Although performance on the ANTA is correlated with divergent thinking, it specifically demands participants to engage in semantic and episodic future thinking, which is not always required in divergent thinking tasks. It is possible that anticipatory thinking of nearer future (thus less future-orientation) may be more influenced by the three dispositional factors.

A primary limitation of this study was its use of Mechanical Turk workers rather than professional analysts due to challenges in population access. The proposed ANTA also comes with some limitations. The ANTA was primarily developed around the extrapolation component of anticipatory thinking, and it is currently unknown how it relates to the two other facets, namely, recognition and construction. Development of proper measures of the two additional facets need before such relations can be explored. A second limitation of the ANTA is its shortened task duration, while ideal for measurement, may not provide the complete picture for anticipatory thinking processes that can take months in some cases. Another potential limitation is that some of the high correlation between the uniqueness, specificity, and remoteness measures could be due to common method variance. Given the use of different raters and the high correlation of these metrics with the diversity metric, which used a different method, this concern was partially alleviated. Finally, analyzing data from the ANTA requires labor intensive qualitative coding for each response.

Despite these limitations, we believe the ANTA provides the basis for a valuable assessment for anticipatory thinking. The ANTA provides a flexible method for assessing anticipatory thinking skills in a broad range of domains. An instrument for anticipatory thinking could be used to assess the efficacy of structured analytical methods, research the thought process of anticipatory thinking, and could supplement the current battery of tests in the selection of personnel for occupations that require strong anticipatory thinking skills. Future research is needed to develop an understanding of the process of anticipatory thinking and further evaluation of the ANTA through relating it to professional analysts’ job performance measures. It may also be possible to alleviate the resource burden of evaluating the ANTA responses in the future through the application of natural language processing for automated assessment.

## Conclusion

Anticipatory thinking is an essential skill for many professions that center on navigating uncertain futures. The ANTA provides a method for assessing an individual’s ability to engage in the generative component of anticipatory thinking and generate a corpus of alternative futures. It provides the foundation for tools that can yield deeper insight into the process of anticipatory thinking, identify how support environments can best assist practitioners, and develop training to enable individuals to improve their ability to apply anticipatory thinking skills in the field.

## Ethics Statement

This study was approved by the Institutional Review Board of North Carolina State University in Raleigh, NC, United States, and the Institutional Review Board of the United States Department of Defense.

## Author Contributions

All authors were involved in conceptualizing the study, piloting the initial versions of the task, and writing the manuscript. MG, AS, JC, and JF were involved in collecting the data. MG was involved in analyzing the data. All authors approved the final submitted version of the work.

## Conflict of Interest

The authors declare that the research was conducted in the absence of any commercial or financial relationships that could be construed as a potential conflict of interest.
