# Supplementary material for: Construction and Validation of an Anticipatory Thinking Assessment
Source: Front Psychol. 2019 Dec 11;10:2749. doi: 10.3389/fpsyg.2019.02749 (PMC6917603; doi:10.3389/fpsyg.2019.02749)
Supplement: Supplementary file 1 [file Data_Sheet_1.docx]

**Appendix A**

Anticipatory Thinking Assessment (ANTA) Instructions

In the following sections you will be given a topic and asked to anticipate possible risks, opportunities, trends, or other uncertainties and how they may affect the topic. The goal is to provide creative and unique responses that describe as many possible futures as you can. Do not worry about spelling or grammar.

Some examples of answers for the prompt "Nutritiously and sustainably feeding 8.5 billion people in 2030" can be seen below, along with an evaluation of the response.

| **Uncertainty** | **Impact** | **Evaluation** |
| --- | --- | --- |
| More international trade | Resource efficient food production | Okay: Appropriate but not particularly creative |
| Rapid adoption of new food tech | Increased preference for vegetarianism | Great: Unique impact |
| Increased preference for vegetarianism | Lower resource requirements for production | Good: Creative and chained with previous response |
| Advancement in synthetic meat production | Lower production costs for meat | Okay |
| Large scale warfare | Wartime costs raise production costs decreasing food availability | Good: Unique uncertainty |

To familiarize yourself with the interface, you will first complete a shortened version of the exercise. You will be presented with a prompt and asked to assess your familiarity with the topic. Next, you will have 3 minutes to generate as many uncertainty/impact pairs as you can. Please click next to proceed.

**Appendix B**

ANTA Rating Instructions

*Training Instructions*

When reading through all the uncertainty and impact pairs it would be good to establish as a group **during the training** how everyone rates based on the criteria. One person may tend to be critical, while another is lenient. Consider how you are rating compared to the others and work to find the middle ground definition of a true 1, 3, and 5 answer as a group. This will be helpful if you are ever unsure about a response, thus allowing you a reference to come back to. Note that the more knowledge you have on a subject the more likely you will be to rate it higher, especially on creativity. This works both ways, as something you know little about will lead you to rank it lower. If you do not know a word, **look it up**! Understand that impacts can seem like they are missing information, because they are a response to the uncertainty. You many need to take this into account to understand the scope for judging and ranking it. **Reading through the data first is important**, especially for creativity. If you code a thousand responses, it will be a hassle to go back and recode because a response started to appear more frequently as you went on. Note that this can get tedious, and it helps to focus on nonresponses when you get tired. This allows you to just get something done if you are unable to focus.

*General Rating Instructions*

You will assess answers from individuals who have participated in a survey designed to help measure their anticipatory thinking. Each answer includes an uncertainty--a potential change or state of the future--an impact--a possible result from that uncertainty that the individual thought were potentially relevant for understanding the future of a provided topic.

Successful Anticipatory Thinking identifies a broad range of clear possible futures that planners need to consider, so you will assess each answer in terms of:

1. How original/unique/novel each uncertainty and impact is, on a scale from 1-5.
   - Original/unique/novel answers ensure that the individual has considered a wide range of possible futures. They will occur more rarely in the dataset.
2. How specific/fully elaborated each uncertainty and impact is, on a scale from 1-5.
   - Specific, fully elaborated answers ensure that the individual has clearly defined a future well enough to help a planner think about it.
3. How uncommon/remote/clever it is to associate the impact with the provided uncertainty.
   - Uncommon/remote/clever associations between uncertainties and impacts ensure that the individual is thinking creatively about how the future could unfold.

We will weigh your scores based on how closely you agree with other raters who evaluate the same items and pairs. Please read all answers in each section before assigning your ratings.

*Novelty Instructions*

Use your judgment to assess on a scale of 1-5 how original/unique/novel each uncertainty or impact listed below is. A score of 1 indicates an unoriginal/common/obvious answer and a 5 indicates an original/unique/novel answer. You may enter a score of 0 if the answer cannot be understood.

*Specificity Instructions*

In column "Specificity", please assess on a scale of 1-5 how specific/fully elaborated each uncertainty or impact listed below is. A score of 1 indicates a vague answer and a 5 indicates a specific/fully elaborated answer. You may enter a score of 0 if the answer cannot be understood.

*Remoteness Instructions*

Please read through all of the uncertainty and impact pairs listed below. In this section, use your judgment to assess on a scale of 1-5 how uncommon/remote/clever it is to associate the impact with the provided uncertainty. You may enter a score of 0 if the answer cannot be understood.

*Categorization Instructions*

Categorize each uncertainty and impact listed below according to the list of available categories for each prompt. You may enter a NA if the answer cannot be understood.

*Examples*

Categorization for Leisure Time Task

| Category | Examples |
| --- | --- |
| Economic (macro) | - Decrease in social capital and time spent with friends  - Large venue sites such as Disneyland will just keep jacking up prices with more people on vacations and time off in the next 10 years |
| Emotional Health | - Free massages for everyone once a month  - Spiritual level of the entire nation will be lifted by increased leisure time, thus reducing the amount of mental health issues and revitalizing a shift towards positive psychology |
| Entertainment / Leisure Time | - Senior citizens and leisure time goals  - After the initial blooming a stagnation of ideas occurs. People get a little wild - Think 1920's America. Sex, parties, illicit drugs. |
| Food | - Cooking technology will reduce the amount of effort and time spent cooking  - Increase in local food industries to accommodate tourists |
| Global health (environment) | - Global natural disasters  - Pollution of our outdoor campgrounds and lakes, etc will only expand as our population expands and uses these resources for vacations, etc. |
| Law and Crime | - Terroristm increases  - Ethically questionable or unlawful leisure activities to receive mark of approval in society |
| Mobility | - Travel restrictions  - Greater access to 'leisure' activities from the comfort of home |
| Physical Health | - Increase in helmets and guards, injuries and need for walk-in medical offices  - Healthy lifestyles |
| Political | - Governments instill new ways to collect tax from citizens - now even VR experiences taking place using Government Lands are taxed  - Challenge of key capitalist interest and values |
| Social | - Culture shifts more toward hedonism  - With more and more people on time off and traveling services for the handicapped will be reduced |
| Work | - Work ethics will decline with less leisure time  - As workers compete for less jobs companies will reduce benefits such as vacation |
| Technology | - More technology  - The whole world is pounded down into small outcrops of survivors doing their best to defend against the now rogue robotic soldiers |

Ratings for Leisure Time Task

Example 1

Response: Sex discrimination disappears as the biological clock excuse evaporates

Category: social norms/way of life

Specificity: 5 (Very specific and detailed response)

Creativity: 5 (This response is unique and creative)

Example 2

Response: Keeping electric bills low and being environmentally helpful all in one

Category: Environmental

Specificity: 3 (Specific response but still lacks how electric bills will be kept low)

Creativity: 3 (Creative but the data set has similar ideas. Not the most unique.)

Example 3

Response: Products might be hard to use

Category: tech-adoption/trust

Specificity: 2 (This response gives more than just hard to use. We can see idea that products are hard to use)

Creativity: 1 (Hard to use products is still a common response)
